# Comprehensive Circulatory Metabolomics in ME/CFS Reveals Disrupted Metabolism of Acyl Lipids and Steroids

**DOI:** 10.3390/metabo10010034

**Published:** 2020-01-14

**Authors:** Arnaud Germain, Dinesh K. Barupal, Susan M. Levine, Maureen R. Hanson

**Affiliations:** 1 Department of Molecular Biology and Genetics, Cornell University, Ithaca, NY 14853, USA; ag297@cornell.edu (A.G.); cfssuelev@earthlink.net (S.M.L.); 2 UC Davis Genome Center—Metabolomics, University of California, Davis, CA 95616, USA; dinkumar@ucdavis.edu

**Keywords:** ME/CFS, metabolomics, acyl cholines, steroids, dipeptides, lipids

## Abstract

The latest worldwide prevalence rate projects that over 65 million patients suffer from myalgic encephalomyelitis/chronic fatigue syndrome (ME/CFS), an illness with known effects on the functioning of the immune and nervous systems. We performed an extensive metabolomics analysis on the plasma of 52 female subjects, equally sampled between controls and ME/CFS patients, which delivered data for about 1750 blood compounds spanning 20 super-pathways, subdivided into 113 sub-pathways. Statistical analysis combined with pathway enrichment analysis points to a few disrupted metabolic pathways containing many unexplored compounds. The most intriguing finding concerns acyl cholines, belonging to the fatty acid metabolism sub-pathway of lipids, for which all compounds are consistently reduced in two distinct ME/CFS patient cohorts. We compiled the extremely limited knowledge about these compounds and regard them as promising in the quest to explain many of the ME/CFS symptoms. Another class of lipids with far-reaching activity on virtually all organ systems are steroids; androgenic, progestin, and corticosteroids are broadly reduced in our patient cohort. We also report on lower dipeptides and elevated sphingolipids abundance in patients compared to controls. Disturbances in the metabolism of many of these molecules can be linked to the profound organ system symptoms endured by ME/CFS patients.

## 1. Introduction

Myalgic encephalomyelitis/chronic fatigue syndrome (ME/CFS) is a perplexing disease, silently destroying millions of lives. Indeed, the latest prevalence rate of ME/CFS estimates that there are over 65 million suffering individuals throughout the world [1], and 35–40% of patients are male. Thus, while women are more often diagnosed, the disease is by no means rare in males. Debilitating symptoms of ME/CFS encompass profound fatigue, unrefreshing sleep, post-exertional malaise (PEM), as well as cognitive impairment and/or orthostatic intolerance.

At least half of the 11 major organ systems in the human body appear to be disrupted in ME/CFS patients. Some established instances include the reported dysfunction of several types of immune cells [2,3,4,5], as well as elevated levels of inflammatory cytokines plasma levels in patients compared to healthy controls [6,7]. Neuroimaging has detected a number of abnormalities [8,9,10], and impaired cognitive function is a predominant feature of the disease. Additionally, many patients suffer from digestive problems [11,12], with around 50% of the patient population reporting irritable bowel syndrome (IBS).

The circulatory system is fundamental to all the other systems as it supplies them with necessary compounds while evacuating metabolic waste. The effort to characterize the blood metabolome of ME/CFS patients, with the dual goal of establishing routine diagnostic strategy and gaining further insight into the underlying cause of the disease, has been carried out by several groups on distinct populations of limited size [13,14,15,16,17,18,19,20,21]. The major conclusions of those reports, including ours, generally revolve around differences in amino acids, lipids, and an imbalance in energy and redox metabolisms. Although these studies are informative, as yet no metabolite has been consistently found to be significantly altered in ME/CFS patients compared to healthy controls, a result that is perplexing when considering the severity of disease symptoms.

For this latest study, we provided plasma from 52 female subjects, equally sampled between controls and patients, for analysis by Metabolon^®^’s mass spectrometry. The metabolic panels yielded datapoints for about 1750 blood compounds with a large emphasis on lipids (over 70%). Our analysis provides novel insights into plasma metabolomics in ME/CFS in several promising areas. Primarily, we expose a reproducible disruption in acyl choline metabolism, a class of lipids that remain largely unstudied but are theorized to have fundamental functions. Another key observation, possibly relevant to the nervous system disruptions in the ME/CFS population, involves sphingolipids, which are also widely affected in our patient cohort. We also find, for the second time, that dipeptides are altered. Finally, three steroid classes (androgenic, progestin and cortico-) are consistently reduced in our ME/CFS patient cohort, a disturbance that could have major repercussions on the well-being of patients.

## 2. Results

### 2.1. Population Statistics and Dataset Handling

#### 2.1.1. Cohort Statistics

The female cohort for this study is comprised of 26 healthy controls and 26 ME/CFS patients between the age of 22 and 72 and with comparable body mass index (BMI). Further description of the patient population is detailed in Table 1, including the type of onset, where 58% identified a sudden illness that immediately preceded the onset of the disease, while 42% consider their onsets to be gradual.

Moreover, half of the patients also reported gut inflammation symptoms, and 69% of the patients reported being diagnosed with orthostatic intolerance. The Bell scores reported for the patient cohort attest of the severity of the symptoms endured by the patients, with almost all of them under the score of 50, far from the score of 100 expected of a healthy individual. Furthermore, the SF-36 aggregated summaries (PCS and MCS) for both groups, demonstrate the impairment of the patients, especially with a SF-36 PCS score at 26, well below the US general population norm of 50 [22].

#### 2.1.2. Global Metabolomics Panel

The global metabolomics panel performed on plasma yielded measurements for 768 identified compounds, sub-categorized into nine metabolic super-pathways, per Metabolon^®^’s standards. The partitioning is as follow: amino acids (196), carbohydrates (25), cofactors and vitamins (29), energy (10), lipids (259), nucleotides (33), partially characterized molecules (2), peptides (33), and xenobiotics (181). Across 52 subjects, there are a cumulative 39,936 datapoints, which can be further divided into 94 sub-pathways (Supplementary File 1). For instance, if we focus on the seven sub-pathways within the xenobiotics super-pathway, we can find bacterial/fungal (3), benzoate metabolism (21), chemical (23), drugs (64), food component/plant (49), tobacco metabolites (6), and xanthine metabolism (15).

Such level of detail is crucial when handling missing data either because of the absence of a compound or present in amounts below the threshold of detection of the equipment used. For this dataset, 15.5% of values are missing (6170), similar to the 14% of imputed data for our previous study [20]. The handling of missing data must differ depending on whether a metabolite is required for human metabolism or a substance taken by a few subjects in our study. As such, we assume that missing values for tobacco metabolites and drugs (3138 or 50.9% of missing values) are due to the truly missing compounds in the blood of those subjects and can be imputed as zero values. On the contrary, other sub-pathways contain metabolites that are biologically required even if not detected. The imputation strategy was therefore different and followed recommendations from Metabolon^®^ to assign the minimum value for each biochemical within the project. Out of the remaining 698 metabolites, 418 were measured in all subjects, 163 had missing values in less than 20% of subjects and 657 imputations were performed (10.5% of missing values), while 117 metabolites had missing values in more than 20% of the subjects for a total of 2375 imputations (38.4%). The level of imputation is similar for each cohort.

#### 2.1.3. Complex Lipid Panel^TM^

In contrast to relative amounts reported in the global metabolomics panel, the complex lipid panel^TM^ generated concentration data for 1007 complex lipids across 11 super-pathways and 19 sub-pathways, per Metabolon^®^’s nomenclature (Supplementary File 2). This included the following super-pathways: cholesterol esters (26), diacylglycerols (58), free fatty acids (25), lysophosphatidylcholines (18), lysophosphatidylethanolamines (17), monacylglycerols (26), phosphatidylcholines (103), phosphatidylethanolamines (127), phosphatidylinositols (28), sphingolipids (61), and the largest one, triacylglycerols (518). This dataset had 3472 missing datapoints (50.7% in controls and 49.3% in patients), mainly from the following super-pathways: lysophosphatidylethanolamines, phosphatidylcholines, phosphatidylethanolamines, and phosphatidylinositols, which had 23, 20, 37, and 14% of missing data respectively. The minimum value replacement strategy described for the global metabolomics panel was identical for this dataset.

### 2.2. Statistical Analysis of the Global Metabolomics Dataset

#### 2.2.1. Whole Dataset Analysis

- Non-parametric tests

A non-parametric Wilcoxon rank-sum test found 41 metabolites out of 768 (5.3%) to be significantly different between controls and patients at *p* < 0.05 (Table S1). Only eight of those (20%) have higher abundance in the patient cohort compared to the controls. Over 60% belongs to the lipids super-pathway, 20% are amino acids, and the remaining 20% was equally divided between peptides and xenobiotics. However, after adjustment for multiple comparison testing (FDR), none were below a *q* = 0.15 cutoff, all had the same value at *q* = 0.64.

- Fold change analysis

A fold change analysis between the two cohorts finds 25 metabolites to have absolute values changes with a threshold of two (Table S2). Only five of those (20%) show higher measurements in the patient cohort compared to the controls. Half of the 25 metabolites are classified as xenobiotics where differences can be traced to a few outlier subjects, sometimes in controls and other times in patients (Supplementary File 1).

**Figure 1:**
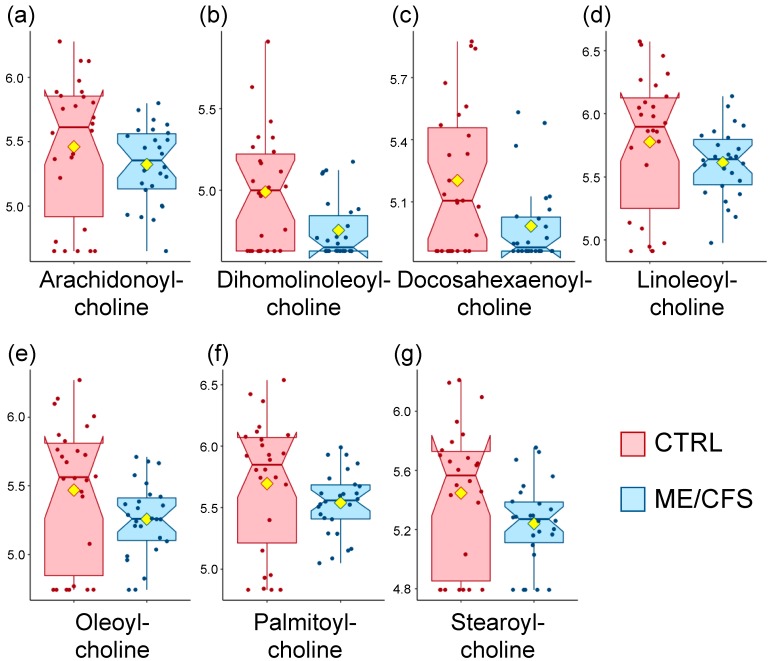
Box plot distribution of logged values for the metabolites that are part of the acyl choline pathway. Controls (CTRL) are shown in red and patients (myalgic encephalomyelitis/chronic fatigue syndrome (ME/CFS)) in blue. The yellow diamond represents the mean. (**a**) Arachidonoyl-choline, (**b**) Dihomolinoleoyl-choline, (**c**) Docosahexaenoyl-choline, (**d**) Linoleoyl-choline, (**e**) Oleoyl-choline, (**f**) Palmitoyl-choline, (**g**) Stearoyl-choline.

Most noteworthy is the presence of several lipids belonging to the sub-pathway of fatty acid metabolism (acyl cholines). Indeed, the means of oleoylcholine, dihomo-linoleoyl-choline, linoleoylcholine, stearoylcholine, and palmitoylcholine were more than twice as low in patients compared to controls. Additionally, Figure 1 shows that the data distribution of all of the seven metabolites belonging to this sub-pathway is lower in the patient cohort compared to the controls. In fact, it is the same few subjects in the patient cohort that are over the median value of the control cohort. The data imputation is comparable between each cohort and this trend remains the same when missing datapoints are ignored instead of being imputed with the minimum value strategy (not shown).

The other lipid reduced by over three-fold in the patient cohort is cholate, a major primary bile acid produced in the liver and known to facilitate fat absorption because of its detergent properties [23]. All the other metabolites classified in that sub-pathway were also lower in the patient cohort compared to the control cohort at a 1.3 to 1.6-fold (Table S2).

Similarly, pyridoxate is reduced by over three-fold in the patient cohort. The only other measured metabolite belonging to the Vitamin B6 sub-pathway is pyridoxal and it was also reduced in patients by 2.3-fold (Table S2).

- Volcano plot analysis

A volcano plot analysis combines fold change and Wilcoxon rank-sum test. Here again, the use of *q*-values does not yield any significantly different metabolites between controls and patients. However, when fold change is combined with a *p* < 0.1, five out of the ten metabolites deemed significant belong to the acyl choline pathway, namely, dihomo-linoleoyl-choline, oleoylcholine, stearoylcholine, linoleoylcholine, and palmitoylcholine (Table 2).

Four of the other metabolites are xenobiotics linked to diet choices. For instance, erythritol is a non-caloric sweetener, generally more abundant in patients with the exception of one strong control outlier (Table 2 and Figure S1c); piperine and its sulfated form are linked to pepper consumption and less abundant in patients who most likely avoid spiced food because of digestive problems (Table 2 and Figure S1d,e); while dimethyl sulfone is a known plasma organic sulfur compound partially derived from intestinal bacterial metabolism, which is perturbed in ME/CFS patients (Table 2 and Figure S1b) [24]. Finally, 4-hydroxyglutamate was found to be twice as high in patients compared to controls (Table 2 and Figure S1a). This latter metabolite is part of the glutamate metabolism, which has substantial implications in brain function [25].

#### 2.2.2. Super-Pathway Dichotomized Analysis

As performed in our previous study, based on the super-pathway nomenclature provided by Metabolon^®^, we further split our dataset before generating the *q*-values, with the goal of regaining statistical power in a large dataset from a limited sized population [20]. The peptides super-pathway contains three dipeptides that are significantly lower in patients compared to controls (Table S3). Dipeptides are incomplete breakdown products of protein digestion/catabolism and some are known to have an effect on body functions [26,27].

These were the only metabolites that were significantly different between controls and patients with a *q* < 0.15.

### 2.3. Statistical Analysis of the Lipidomics Dataset

**Figure 2:**
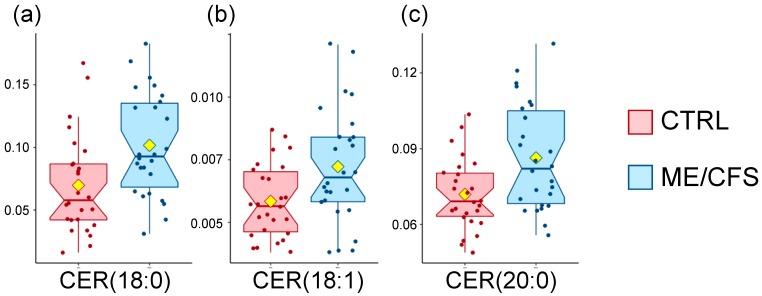
Box plot distribution of concentrations for the metabolites in Table 3. Controls (CTRL) are shown in red and patients (ME/CFS) in blue. The yellow diamond represents the mean. (**a**) CER(18:0), (**b**) CER(18:1), (**c**) CER(20:0).

A non-parametric Wilcoxon rank-sum test finds three metabolites out of 1007 to be significantly different between controls and patients at *p* < 0.05 (Table 3). All three belong to the sphingolipids super-pathway, namely the following ceramides, CER (18:0), CER (18:1), and CER (20:0), had higher levels in the patient cohort compared to the controls (Figure 2). Ceramides are made of a sphingosine (d18:1) amino acylated at the 2-carbon position to a long chain fatty acid, and while they make up a majority of the cell membrane’s lipid bilayer, they are particularly abundant in the myelin sheath. They also contribute to a variety of signaling such as cell differentiation, proliferation, and cell death [28].

A fold -change analysis yields five compounds that are all part of the triacylglycerol pathway, but careful scrutiny reveals that it is due to one extreme outlier within the controls and a couple less extreme outliers within the patient population. Those subjects are specifically outliers for a number of the triacylglycerols (TAG) analyzed, but not all (Supplementary File 2), and cannot be considered as outliers for our datasets.

A volcano plot analysis does not reveal any difference between controls and patients.

### 2.4. Metabolomics Data Insight

In an effort to pursue biological relevance, it is crucial to interpret our datasets from a different and more comprehensive perspective. To this end, we used MetaboAnalyst Pathway Enrichment as well as ChemRICH (a Chemical Similarity Enrichment Analysis for Metabolomics) to further explore our data.

#### 2.4.1. Pathway Enrichment

- Global metabolomics panel

For this MetaboAnalyst module, compounds are mapped to defined pathways based on their HMDB IDs (Human Metabolome Database). Out of the 768 metabolites analyzed by Metabolon^®^, 527 had such ID, and 477 were suitable after ID standardization. As a result, 38% of the metabolites were not part of this analysis, including all of the compounds that are part of the acyl choline pathway (Table 2 and Figure 1), for example. Some super-pathways were more affected than others, with ~50% of peptides and xenobiotics metabolites absent, ~40% of energy and lipids metabolites absent and ~20% of the metabolites in the remaining super-pathways.

Identical filtering and normalization steps were performed as described in the experimental section on this reduced HMDB dataset and the output includes 61 pathways (Table S4), but only three have significant enrichment at *p* < 0.05, namely glutathione metabolism, steroid hormone biosynthesis, and propanoate metabolism. However, none of them are significantly enriched after multiple testing correction (*q*-value) and their impact score is very low, below 0.08 (Table S4). Four pathways had a relatively high impact score (above 0.4), but a *p* > 0.11: alanine, aspartate, and glutamate metabolism, taurine and hypotaurine metabolism, caffeine metabolism and linoleic acid metabolism (Table S4). While a few of those pathways have been discussed in published work and can be linked to redox imbalance and amino acid metabolism for example, none of them meet the triple criteria of a high impact score as well as significant *p* and *q*-values.

- Complex lipid panel^TM^

Out of the 1007 lipids analyzed by Metabolon^®^, 392 have an HMDB ID and 380 were included in the pathway enrichment analysis, only 38% of the dataset. The output includes eight pathways (Table S5), and the sphingolipid metabolism pathway has an impact score of 0.34 and a *p*-value of 0.03. While the *q*-value is at 0.23, this is the only pathway that meets two criteria out of three. We mention sphingolipids as part of the lipidomics dataset analysis (Figure 2 and Table 4).

In this module, all compounds of importance are more abundant in patients compared to controls, similarly to the discussed ceramides in Figure 2 and Table 4. The ones with most importance are sphingomyelins (KEGG = C00550), followed by ceramides (KEGG = C00195), and finally glucosylceramides (KEGG = C01190) as well as lactosylceramides (KEGG = C01290) (Table 4). It is important to note that each KEGG number above encompasses from eight to nine compounds each, structured as a sphingosine (d18:1) and fatty acid chains including saturated or unsaturated C16, C18, C20, C22, C24, and C26. Ceramides, also known as N-acylsphingosine, are one of the hydrolysis byproducts of sphingomyelins. Glucosylceramides and lactosylceramides are involved in numerous pathways and dysfunctions occurring in several diseases [29,30].

#### 2.4.2. Statistical Enrichment

- Global metabolomics panel

Unlike pathway mapping, the ChemRICH approach relies on structure similarity and chemical ontologies to map study-specific metabolites. To achieve this, one of the identifiers used is Pubchem ID that labels chemical molecules and their activities in biological assays. The information required for this online tool included the compound name, the InChiKeys (International Chemical Identifier), the Pubchem ID, the SMILES identity (Simplified Molecular Input Line Entry System), which is a line notation for both entering and representing molecules, and finally *p*-values from the Wilcoxon rank-sum test as well as fold changes (outputs were similar between fold changes of medians and means; fold change of means were used in the results discussed). Out of the 768 metabolites with data from Metabolon^®^, 623 were suitable for analysis. This means that 19% of the metabolites were not part of this analysis, and this time, three of the compounds classified as part of the acyl choline pathway (Table 2 and Figure 1) were included, namely, oleoylcholine, palmitoycholine, and stearoylcholine.

Only one out of the 86 cluster names generated by this analysis is considered significant at *p* = 0.025, although with a *q*-value of 1 (Table S6). The cluster name is labelled as “dipeptides” and contains 52 compounds, nine of which are altered (Table S7). The key compound of this cluster is 4-hydroxyglutamate, mentioned earlier (Table 2 and Figure S1a), and is increased in patients compared to controls as are two other metabolites in this same cluster, namely: hydroxyasparagine and gamma-glutamyltyrosine. On the other hand, six compounds classified as dipeptides by ChemRICH are decreased in patients compared to controls: phenylalanylalanine and phenylalanylglycine (Tables S3 and S7), cysteinylglycine, valylleucine, 3-hydroxylaurate, and etiocholanolone glucuronide (Table S7). All are also present in Table S1 as they have *p*-values < 0.05.

None of the other 85 cluster names are significantly different between controls and patients. However, with *p* = 0.057, the next cluster named “NewCluster_16” contains two acylcarnitines [arachidoylcarnitine (C20) and adipoylcarnitine (C6-DC)] which are more abundant in patients compared to controls.

- Complex lipid panel^TM^

As stated previously, 392 of the lipids have an HMDB ID, which was used to gather the required fields to run the ChemRICH analysis. In the end, 347 were included in the pathway enrichment analysis, only 34% of the dataset. None of the 26 monacylglycerols and none of the 518 triacylglycerols had an HMDB ID and are therefore not included in this analysis.

Two clusters are significantly different out of 20 cluster names (Table S8). The first one denominated “Unsaturated_Ceramides”, with *p* = 0.0004, shows three out of nine compounds that are increased in ME/CFS patients compared to controls, namely: CER (18:0), CER (18:1), and CER (24:1) (Table 5). The first two are already mentioned in Table 3 and Figure 2 while CER (20:0) was not included in this analysis as it does not have the sufficient ID requirements for ChemRICH. The second cluster name is “Sphingomyelins,” with *p* = 0.035, where two out of nine compounds are increased in ME/CFS patients compared to controls, namely: SM (18:0) and SM (18:1) (Table 5). Sphingomyelins are already mentioned as part of the MetaboAnalyst pathway analysis results.

According to Metabolon^®^’s nomenclature, all five metabolites discussed here are part of the super-pathway sphingolipids, which were first discovered in brain extracts in the 1870s, and have a severe impact on neural tissue when imbalance occurs [30].

- Sub-pathway-based enrichment analysis

To circumvent the limitations described above, a sub-pathway-based analysis according to the sub-pathways provided by Metabolon^®^ was performed using the ChemRICH concept. This analysis combined both complete datasets to look for difference in patterns between our control and patient cohorts, according to sub-pathway classification, *p*-values and fold changes. Six of the seven significantly (*p* < 0.05) impacted metabolite clusters were not classified as drugs and all are displayed in Figure S2. Those six clusters are androgenic steroids, acyl cholines, ceramides, dipeptides, acyl carnitines, and sphingomyelins. Some details of this analysis are shown in Table 6 and further details can be found in Table S9. All of those clusters appeared throughout our analysis when only *p*-values were considered, but for the first time, three remain significant after correction (*q* < 0.05), and the first five if we apply a *q* < 0.15 (Table 6). The trend within each cluster is unique, with all of the altered androgenic steroids and acyl cholines decreased in patients compared to controls, all of the altered ceramides increased, while five out of the eight altered acyl carnitines are decreased (Table 6 and Figure S2 where colors reflect such patterns). Corticosteroids are another class of steroids and were the next significant cluster, even though they are not part of Table 6 because of a *p*-value at 0.056 (*q* = 0.77). Similar to androgenic steroids, both of the altered corticosteroids are decreased in patients compared to controls.

#### 2.4.3. Inclusive Observation of the Metabolomics Data

One striking observation is that no broad difference is observed when comparing the control cohort to the patient cohort. This is, by itself, a result worth noting that we will discuss later. Nevertheless, it is also vital to reflect on the methods used and their limitations, especially in light of the numerous variables ignored (from 19 to 66%) by both MetaboAnalyst and ChemRICH. Although the ChemRICH sub-pathway-based analysis provides valuable and statistically sound insight into our dataset, we opted to design simple but equally comprehensive calculations to analyze our datasets with less stringent statistical rigor in order to enhance our ability to fully understand the metabolomics status of ME/CFS patients. Therefore, we decided to compute the ratio (controls/patients) of the median for each metabolite analyzed by Metabolon^®^ (this data is provided as an extra column in Supplementary Files 1 and 2). The median was chosen over the mean as some metabolites span a wide range of abundance and some means proved to be biased when no further statistical consideration was applied as in other methods used throughout this manuscript. We further grouped the metabolites in accordance with Metabolon^®^’s sub-pathway divisions, by averaging the ratio of the medians for all compounds of a given sub-pathway, resulting in 94 datapoints for the global metabolomics panel (Table S10). The same process was implemented using the complex lipid panel^TM^ (data not shown), with no differences to report.

**Figure 3:**
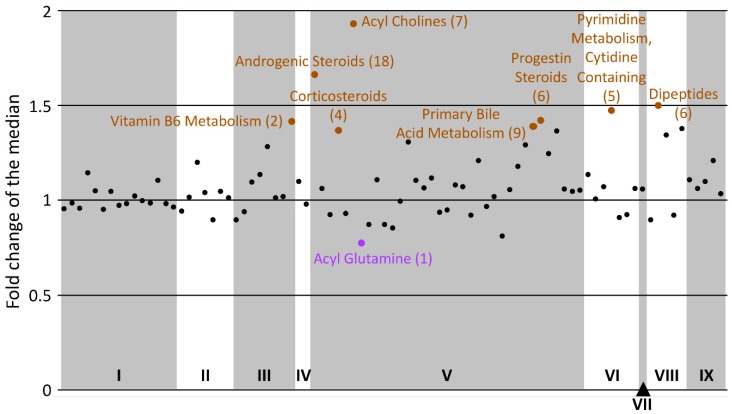
Display of the fold change (controls/patients) of the median, averaged for each of the 94 sub-pathways. Roman numbers at the bottom of the figure are assigned as follow for each of the nine super-pathways: I = amino acids, II = carbohydrates, III = cofactors and vitamins, IV = energy, V = lipids, VI = nucleotides, VII = partially characterized molecules, VIII = peptides, and IX = xenobiotics. Labelled sub-pathways are discussed in the manuscript; brown ones are over-abundant in controls compared to patients while purple ones are the opposite. The number associated with each sub-pathway reflects the number of metabolites included. Omitted from the graph are eight drug sub-pathways as well as the tobacco metabolites, all classified as xenobiotics.

Figure 3 contains 85 of the datapoints displayable and clearly shows that within the nine super-pathways, the majority of the sub-pathways have an averaged fold change of the median close to 1, reflecting no general dysregulation between the control and the patient cohorts. Nevertheless, a few sub-pathways stood out because a majority if not all of their metabolites were dysregulated in the same direction for a cohort versus the other.

The most obvious alteration is acyl cholines with a ratio of 1.9 (Figure 3). As depicted in Figure 1, all of its members are lower in the ME/CFS cohort compared to the control cohort, and five of them are significantly different according to our volcano plot analysis (Table 2).

This analysis also brings attention to androgenic steroids (ratio = 1.7). Here again, all members of this sub-pathway are lower in ME/CFS versus controls. Seven of them are present in Table S1 with *p*-values < 0.05, even though we initially ignored them based on multiple testing correction. Androgenic steroids are already highlighted in the ChemRICH analysis (Table 5 and Table 6). All 18 metabolites have reduced abundances in ME/CFS, with ratios varying from 1.1 to 4. Androgenic steroids include DHEA (dehydroepiandrosterone) and are abundant circulating steroids with varying biological functions, including important effects on the central nervous system [31]. Two other steroid pathways also appear to be downregulated in ME/CFS, progestin steroids and corticosteroids. Out of the six metabolites of the first sub-pathway, five had a ratio between 1.3 and 1.7 and the sixth one was at 0.94. Progestin steroids are more narrowly involved in the female menstrual cycle. On the contrary, corticosteroids are involved in an extremely wide range of physiological processes, including but not limited to immune response, inflammation regulation, and protein catabolism [32]. While one had a ratio of 0.98, the other three were at 1.36, 1.46, and 1.65 between our two cohorts of 26 female subjects each.

Dipeptides were also part of the sub-pathways with a high averaged ratio of fold change medians. Six dipeptides are in our dataset, and four of them have been mentioned throughout our data analysis in Table 6, Tables S3 and S7. Valylglycine did not come up as it has a median fold change of 1 and neither did leucylglutamine, even though it has a fold change of 1.48. Overall, the dipeptide sub-pathway is upregulated in ME/CFS patients.

Two other sub-pathways should be mentioned: pyrimidine metabolism (cytidine containing) and the vitamin B6 metabolism as those are recurring results in the ME/CFS metabolomics literature [18,20] and mentioned earlier (Table S2).

Finally, one compound classified as an acyl glutamine (hexanoylglutamine) had the lowest ratio at 0.77. A higher concentration of this metabolite in patients would indicate an increase in mitochondrial fatty acid β-oxidation (FAO) and could affect energy levels in patients. Such changes have also been linked to Parkinson’s disease although not as a sole biomarker [33].

## 3. Discussion

### 3.1. Acyl Cholines Are Decreased

This class of compound is extremely intriguing in the context of ME/CFS. Although this is only the second time that acyl cholines have been assessed in ME/CFS, the results are reproducible between two separate cohorts. Our previous study [20] contains data for four acyl cholines (arachidonoylcholine linoleoylcholine, oleoylcholine, and palmitoylcholine), and all of them are also reduced in the patient cohort compared to controls (Figure S4). Although none of them are significantly different after correction (*q*-values above 0.4), their p-values and fold change are comparable to the data presented in Table 2 (Table 7).

Acyl cholines are still an obscure class of compounds first described in 1911, in conjunction with blood pressure, where a remarkably extensive study found that short-chain choline esters had a depressor effect while long-chain ones tended to have a pressor effect [34]. Those findings were further corroborated by several groups between 1914 and 1956. At that time, the pharmacological effects of acyl cholines was demonstrated on the isolated rabbit heart, isolated guinea-pig ileum, and the rat stomach [35]. The compounds measured in our datasets, considered long-chained acyl cholines, were found to block the effect of acetylcholine on the rabbit and guinea-pig tissues tested, and depressed spontaneous hydrochloric acid secretion by the rat stomach. Blood pressure problems are one of the many symptoms encountered by ME/CFS patients [36], especially when considering the common dysfunction in orthostatic intolerance (Table 1). A decrease in long-chained acyl cholines could explain a disruption in blood pressure regulation, manifested by dizziness, lightheadedness, blurred vision, and near syncope when assuming and maintaining the upright position. We also know that patients are affected by irritable bowel syndrome [36], among many other intestinal disruptions (Table 1). A decrease in long-chained acyl cholines could disrupt hydrochloric acid secretions with consequences that could be as far reaching as leaky gut symptoms and altered gut microbiome populations. Finally, and although acetylcholine is not one of the metabolites measured in either of our datasets, we note that long-chained acyl cholines have antagonistic activity toward acetylcholine. Acetylcholine was the first neurotransmitter to be identified and is known to be crucial in both the central nervous system (CNS) and the peripheral nervous system (PNS). In the CNS, it supports the cognitive functions of the brain, involving the acquisition of knowledge and understanding through thoughts, experiences, and senses. Those are all skills ME/CFS patients report as diminished [36]; their cognitive issues, often described as “brain fog,” include concentration problems, dyscalculia, memory loss, spatial disorientation to only name a few. In the PNS, acetylcholine acts at the neuromuscular junctions. Acetylcholine functions in the autonomic nervous system, both as an internal transmitter for the sympathetic nervous system (SNS), also called “fight or flight” system, and as the final product released by the parasympathetic nervous system (PSNS), also called “rest and digest” system. Indeed, apart from the numerous digestive problems mentioned above, the hallmark symptom of ME/CFS is overwhelming fatigue that is not improved by rest and is exemplified after any activity, described as post exertional malaise (PEM).

Acyl cholines have been vastly neglected since the 1950s. Linoleoylcholine, oleoylcholine, palmitoylcholine, and stearoylcholine have been sparingly discussed as enhancers of drug absorption from the gastrointestinal tract while a pharmacological experiment found arachidonoylcholine to exhibit cholinomimetic activity, which stimulate the PSNS. Mostly because of Metabolon^®^’s expertise, acyl cholines have recently been mentioned in a few nutrition metabolomics studies on both plasma and serum. In one case, the effect of coffee consumption on the blood metabolism of subjects was evaluated, and while six acyl cholines were found to be significantly associated with coffee intake, there were an additional 99 unrelated metabolites with the same association [37]. As expected, caffeine was one of them, with a fold change up to 73; however, our cohort does not show any difference between controls and patients (1.1-fold-change, Supplementary File 1). Similar results were reported by the same group when focusing on lipidomics [38]. In another case, rice bran and navy bean consumption influenced 71 plasma compounds compared to controls, including a few acyl cholines [39]. Here again, none of the other metabolites measured behaved similarly in our dataset, hinting that the almost exclusive acyl choline differences between controls and ME/CFS patients are not due to nutrition differences but instead specific to pathways related to acyl cholines. Another study, on a different population, limited to rice bran consumption, leads to the same conclusion [40].

### 3.2. Dipeptides Are Decreased

Dipeptides are another class of compounds found to have decreased levels in our patient cohort, both by statistical test (Table S3) and ChemRICH analysis (Table 6 and Table S7), as shown in Figure 3. Our understanding of variations in plasma dipeptide levels is still limited but we note that most of the dipeptides assessed in our previous study, albeit not the same ones, were also less abundant in the patient cohort than in the controls. If we focus on the latest literature and ME/CFS patient symptoms, our attention is drawn toward the therapeutic potential of some dipeptides on fatigue, aging, and muscle recovery [41,42,43].

### 3.3. Sphingolipids Are Increased

Ceramides and sphingomyelins were highlighted in several instances, both with significant increases detected through statistical analysis (Figure 2 and Table 3) and enrichment analysis on the complex lipid panel^TM^ dataset using both MetaboAnalyst and ChemRICH (Table 5, Table 6, Tables S5 and S8). Pathway abnormalities have already been described for sphingolipids in ME/CFS patients, but the results are inconsistent. In a similarly sized cohort, Naviaux and colleagues found ceramides and sphingomyelins to be decreased in the patient cohort, regardless of the gender [16]. This result was not reproduced by Nagy-Szakal and colleagues, where they did not see a consistent decrease in ceramide levels but reported on decreased levels of longer ceramides in patients without IBS [18]. Our dataset instead shows an increase in ceramide levels for our patient cohort compared to controls. When splitting our patient cohort based on their gut symptoms, ceramide levels for patients without gut symptoms tend to be even further increased for CER (18:0), CER (18:1), and CER (20:0).

The inconsistencies reported between studies of ceramide metabolism, and more generally sphingolipids, points to a complicated landscape in ME/CFS. Their prevalence in cell membranes, with their involvement in many cell processes, as well as the numerous unrelated pathologies linked to an imbalance in their levels [30], suggest that changes in sphingolipids are not directly linked to ME/CFS, but are rather a consequence of the disease and the life style that it inflicts on patients such as disturbed diets, lack of physical activity, and medication regimes.

### 3.4. Three Classes of Steroids Are Decreased

The steroids discussed below are produced in the adrenal cortex, the gonads, and the placenta. They play a crucial role in regulating water and salt balance, metabolism and stress response, as well as sexual differentiation and reproduction [44]. In our analysis, we find three categories of steroids to be on average less abundant in ME/CFS patients compared to controls (Figure 3 and Figure S2). Eighteen androgenic steroids were measured, including a few dehydroepiandrosterone (DHEA) derivatives but none of them are the most abundant circulating one. DHEA is known to have a variety of biological effects ranging from intermediate to the biosynthesis of sex steroids to directly binding to cell receptors and acting as a neurosteroid [45]. Three of the four corticosteroids measured (cortisol, cortisone and corticosterone), were lower in patients. They have roles in stress response, memory, and inflammation. Finally, five out of the six progestin steroids measured were also lower in patients. This category of steroids is primarily linked to the female reproductive system with known consequences when imbalances occur, e.g., monthly pain women encounter when this system resets, or during menopause.

The use of steroids to try to alleviate the symptoms of ME/CFS patients has been attempted many times with noticeable improvements for some patients [46]. The medical consensus, however, is that the multitude of risks and side effects brought by these powerful metabolites outweighs their benefits. Nevertheless, some research suggests that ME/CFS is correlated with a reduced function of the hypothalamic-pituitary-adrenal (HPA) axis and a hypersensitivity of the CNS [47]. The role of the HPA axis is not yet fully understood because it regulates so many functions of our bodies, including energy usage, temperature, immune system, digestion, mood, and sexuality. In the context of steroids, the implications of the HPA axis in ME/CFS is wide-ranging and the reduced abundance of three classes of steroids could be linked to a majority of the symptoms and systems affected in ME/CFS patients.

### 3.5. Acyls and ME/CFS

Acyl groups are a moiety containing a double-bonded oxygen atom and an alkyl group (RCO–), meaning that the acyl group is attached to a larger molecule as is the case in all the acyl compounds highlighted throughout this analysis. While the acetyl group is a sub-type of acyl, no acetyl molecule was found different between controls and patients. Instead, we uncovered a class of lipids belonging to the acyl choline sub-pathway as one of the rare compounds that were consistently lower in ME/CFS patients compared to controls. This prompted us to duplicate the analysis displayed in Figure 3 on our previously published dataset [20] (Figure S3). While tocopherol metabolism and ketone bodies were originally discussed, the other four are newly emphasized. Among them are acyl cholines and acyl glutamine which are common between both Figure 3 and Figure S3, while monoacylglycerols (Table S11) is an additional acyl-related sub-pathway that emerge from this analysis. Monoacylglycerols are not part of the dataset released with this latest analysis. The disaccharides and oligosaccharides sub-pathway is part of the carbohydrate super-pathway and refers solely to sucrose, which was lower in patients compared to controls. Acyl carnitines are also found altered in Figure S3.

We have very limited knowledge of both the synthesis pathway and the functions of the majority of these compounds, but they are part of the lipids super-pathway and some links can be found between them. For example, monoacylglycerols are released from diacylglycerols by various lipases, including hormone sensitive lipases which in some cases are involved in steroid production. Another example is the length of the fatty acid chain of virtually all compounds highlighted, between 16 and 28 carbons.

### 3.6. Reproducibility

In our previous study, blood processing was done differently, with blood tubes shipped overnight from a collecting location in New York City, compared to more rapid processing for the samples in this study [20]. Keeping live cells in a self-contained environment for an average delay of 24 h must have affected the amounts of some metabolites, potentially explaining differences in some metabolites between studies. Cells incubated overnight in plasma likely release into plasma, or absorb from plasma, a variety of metabolites. Our current study is more representative of the metabolites circulating in blood of ME/CFS subjects and controls. Results between our two studies can also not be precisely compared because not all the same metabolites were detected in both analyses achieved by Metabolon^®^. For instance, 458 metabolites with an HMDB ID are common between studies, with 25% of the data from Germain and colleagues [20] not present in this dataset, and conversely, 13% of the data in this dataset not present in Germain and colleagues [20].

## 4. Materials and Methods

### 4.1. Cohort and Blood Sampling

Out of the 52 female subjects who participated in this study, 26 were healthy controls, while the remaining 26 were established patients of a ME/CFS specialist in New York City (NYC), Susan Levine, M.D. All patients had a confirmed and rigorous diagnosis of ME/CFS according to the CDC criteria [48]. Healthy controls were selected from healthy female volunteers living in the same geographical area. An effort was made to match age and BMI.

Both ME/CFS subjects and healthy controls completed the medical outcomes survey short form-36 (SF-36) [49] and the Bell disability scale for assessment of severity of symptoms and functional status measure. The physical (PCS) and mental (MCS) component summary scores were calculated from the eight multi-item scales scores of the SF-36 questionnaire [50].

Collection of whole blood, from an antecubital vein with subjects in the seated position, was achieved directly in 8.5 mL BD^TM^ P100 tubes (www.bdbiosciences.com, San Jose, CA, USA) at Dr. Levine’s office. Tubes were transported within two hours, at room temperature, to the NYC Cornell Weill Medical School, where plasma was produced before storage at −80 °C. Shipment of plasma to Cornell’s Ithaca campus was done on dry ice.

### 4.2. Metabolomics Panels

Plasma samples were thawed and 400 μL of each sample was shipped frozen on dry ice to Metabolon^®^’s facility in NC for analysis (www.metabolon.com). The two metabolomics solutions selected included the global metabolomics and the lipidomics panels, resulting in two distinct datasets provided as excel tables.

### 4.3. Data Analysis

Two online services were used to analyze the data. The first one is MetaboAnalyst (www.metaboanalyst.ca) for its statistical analysis and pathway analysis modules [51]. After uploading, data filtering was performed in the following order: interquantile range (IQR), relative standard deviation (RSD = SD/mean), and non-parametric relative standard deviation (MAD/median). Log transformation and auto scaling (mean-centered and divided by the standard deviation of each variable) were used to normalize the data.

The second one is ChemRICH (http://chemrich.fiehnlab.ucdavis.edu) for its statistical enrichment approach [52]. The R-script for the sub-pathway-based analysis is available at www.github.com/barupal. Additional analyses were performed using Microsoft Excel.

### 4.4. Data Availability

The datasets generated for this cohort are available for download as Supplementary Files 1 and 2.

### 4.5. Study Approval

All subjects gave their informed consent for inclusion before they participated in the study. The study was conducted in accordance with the Declaration of Helsinki, and the protocol was approved by the Ethics Committee of Cornell University (IRB 1303003741).

## 5. Conclusions

We are reporting on the largest number of metabolites in the ME/CFS field to date, about 1750 plasma compounds, encompassing 20 super-pathways and 113 sub-pathways. However, we have not unequivocally identified a plasma biomarker or set of biomarkers with abundances drastically different between controls and ME/CFS patients, despite the fact that our clinical data indicates our patient cohort had a substantial level of disability (Table 1). The same conclusion can be drawn from examining various groups’ prior reports, including ours, where distinct populations were recruited, alternate instrumentation was used, or even serum was analyzed instead of plasma [13,14,15,16,17,18,19,20,21].

Nevertheless, the metabolites emphasized as a result of our analysis, most specifically acyl cholines and steroids, should be considered in light of the metabolic impact even modest changes can have along with the complexity of sources that drain metabolites into the circulatory system. Indeed, the changes observed might result from a disturbance occurring in another part of the body (e.g., brain, muscles), one whose impact may be diluted in plasma. Acquisition of samples in conditions that increase patient symptoms (e.g., PEM) could further widen the observed gap between the plasma metabolome of patients and controls. Because liquid biopsies such as plasma and serum remain the most accessible resource from human subjects, our results combined with more explorative metabolomics will help stimulate our efforts before we move toward different, yet more invasive sample collection techniques. Furthermore, set-enrichment statistics with a control for false discovery rates can be used to identify chemical groups instead of individual metabolites that can be associated with a disease or phenotype. Follow-up studies can develop new targeted analytical methods to measure those chemical groups in studies with power calculations from this discovery stage data.

## Figures and Tables

**Table 1:** Details of the population statistics.

		Controls	ME/CFS	Mann-Whitney U Test
**Gender (n)**	Female	26	26	ND
**Age**	Mean +/− SD	41.5 +/− 15	49.7 +/− 13.7	*p* = 0.05
	Median +/− SD	43 (22–66)	52 (22–72)	
**BMI**	Mean +/− SD	21.9 +/− 3.2	24.6 +/− 5.6	*p* = 0.8
	Median +/− SD	21.4 (16.3–28.9)	23 (16.8–40.7)	
**Type of onset**	Gradual	ND	42%	ND
	Sudden	ND	58%	ND
Gut symptoms *		4%	50%	ND
Positive tilt table test ** (*n* = 16)		ND	69%	ND
Bell’s disability scale ***	10–20	0	11	*p* < 0.001
	30–40	0	11	
	50–60	1	4	
	90–100	25	0	
SF-36 ***	Physical Component Summary (PCS)	55.5 +/− 5.3	25.7 +/− 7.8	*p* < 0.001
	Mental Component Summary (MCS)	55.1 +/− 6	40.6 +/− 10.9	*p* < 0.001

* Subjects reporting either irritable bowel syndrome (IBS), ulcerative colitis or Crohn’s disease. ** A tilt test had previously been performed on 16 of the ME/CFS patients, and 11 were positive. *** Higher scores represent better health. ND: not determined.

**Table 2:** Summary of volcano plot analysis with a fold change of mean (controls/patients) threshold of 2 and *p* < 0.1.

Super-Pathway	Sub-Pathway	Metabolite	HMDB ID	Fold Change	*p*-Value
Amino-Acids	Glutamate Metabolism	4-hydroxyglutamate	HMDB01344	0.5	0.005
Lipids	Acyl Cholines	Dihomo-linolenoyl-choline	NA	2.3	0.02
		Linoleoylcholine	NA	2.2	0.07
		Oleoylcholine	NA	2.3	0.04
		Palmitoylcholine	NA	2.1	0.07
		Stearoylcholine	NA	2.2	0.04
Xenobiotics	Chemical	Dimethyl Sulfone	HMDB04983	2.7	0.03
	Food Component/Plant	Erythritol	HMDB02994	6	0.09
		Piperine	HMDB29377	3	0.03
		Sulfate of piperine metabolite C16H19NO3 (3)	NA	2.5	0.03

HMDB stands for Human Metabolome Database. NA stands for not assigned.

**Table 3:** List of metabolites found to be significantly different between controls and patients after Wilcoxon rank-sum testing on the complex lipid panel dataset, with *p* < 0.05.

Super-Pathway	Sub-Pathway	Metabolite	HMDB ID	*p*-Value	Fold Change
Sphingolipids	Ceramides	CER(18:0)	HMDB04950	0.01	0.7
		CER(18:1)	HMDB04948	0.03	0.8
		CER(20:0)	HMDB04951	0.004	0.8

HMDB stands for Human Metabolome Database. Fold change of mean represents controls/patients.

**Table 4:** List of metabolites highlighted as part of the pathway enrichment analysis of the complex lipid panel dataset.

Metabolite Class	KEGG ID	Importance	*p*-Value
Sphingomyelins	C00550	0.01	0.007
Ceramides	C00195	0.29	0.02
Glucosylceramides	C01190	0.03	0.5
Lactosylceramides	C01290	0	0.2

KEGG stands for Kyoto Encyclopedia of Genes and Genomes.

**Table 5:** ChemRICH output for the complex lipid panel cluster name.

Super-Pathway	Sub-Pathway	Metabolite	HMDB ID	Fold Change	*p*-Value
Sphingolipids	Ceramides	CER (18:0)	HMDB04950	0.7	0.01
		CER (18:1)	HMDB04948	0.8	0.03
		CER (24:1)	HMDB06728	0.9	0.02
	Sphingomyelins	SM (18:0)	HMDB01348	0.8	0.008
		SM (18:1)	HMDB12101	0.8	0.003

HMDB stands for Human Metabolome Database. Fold change of mean represents controls/patients.

**Table 6:** ChemRICH output for the sub-pathway-based analysis.

Sub-Pathway	Cluster Size	*p*-Value	*q*-Value	Altered	Increased	Decreased
Androgenic Steroids	18	1.9 × 10^−8^	2.1 × 10^−6^	11	0	11
Analgesics, Anesthetics	20	5.5 × 10^−7^	0.00003	10	2	8
Acyl Cholines	7	0.00002	0.0007	6	0	6
Ceramides	12	0.00004	0.001	7	7	0
Dipeptides	6	0.005	0.1	4	0	4
Acyl Carnitines	39	0.008	0.1	8	3	5
Sphingomyelins	12	0.03	0.5	4	4	0

Clusters were generated per Metabolon^®^’s sub-pathway classification. Increased and decreased refer to patients vs. controls.

**Table 7:** Statistical analysis of acyl cholines measured by Germain and colleagues [20].

Super-Pathway	Sub-Pathway	Metabolite	HMDB ID	Fold Change	*p*-Value
Lipids	Acyl Cholines	Arachidonoylcholine	NA	1.7	0.03
		Linoleoylcholine	NA	1.7	0.02
		Oleoylcholine	NA	1.6	0.06
		Palmitoylcholine	NA	1.6	0.01

HMDB stands for Human Metabolome Database. NA stands for not assigned. Fold change of median represents controls/patients.

## Supplementary Materials

The following are available online at https://www.mdpi.com/2218-1989/10/1/34/s1. Figure S1: Box plot distribution of logged values for the metabolites in Table 2 that are not part of the acyl choline pathway. Controls (CTRL) are shown in red and patients (ME/CFS) in blue. The yellow diamond represents the mean, Figure S2: ChemRICH sub-pathway-based enrichment plot, Figure S3: Display of the fold change (controls/patients) of the median, averaged for each of 89 sub-pathways from Germain et al. (2018), Figure S4: Box plot distribution of logged values for the metabolites that are part of the acyl choline pathway from Germain et al. (2018). Controls (CTRL) are shown in red and patients (ME/CFS) in blue. The yellow diamond represents the mean, Table S1: List of metabolites found to be significantly different between controls and patients after Wilcoxon rank-sum testing with a *p*-value cutoff of 0.05, Table S2: List of metabolites with a fold change (controls/patients) threshold of 2, Table S3: List of metabolites found to be significantly different between controls and patients after Wilcoxon rank-sum testing on super-pathway dichotomized datasets, with a *q*-value cutoff at 0.15, Table S4: Results of the pathway enrichment analysis on the global metabolomics dataset, Table S5: Results of the pathway enrichment analysis on the complex lipid dataset, Table S6: ChemRICH output for the Global Metabolomics panel analysis, Table S7: ChemRICH output for the dipeptides cluster name, Table S8: ChemRICH output for the complex lipid panel analysis, Table S9: Detailed ChemRICH output for the sub-pathway-based analysis, Table S10: Averaged fold change of the median for each of the 94 sub-pathways, Table S11: Statistical analysis of monoacylglycerols measured in Germain et al. (2018), File 1: OrigScale HD4, File 2: OrigScale CLP.
