# Supplementary material for: Comprehensive Circulatory Metabolomics in ME/CFS Reveals Disrupted Metabolism of Acyl Lipids and Steroids
Source: Metabolites. 2020 Jan 14;10(1):34. doi: 10.3390/metabo10010034 (PMC7023305; doi:10.3390/metabo10010034)
Supplement: Supplementary file 1 [file metabolites-10-00034-s001.zip › metabolites-688200-sup.pdf]

metabolites

MDPI

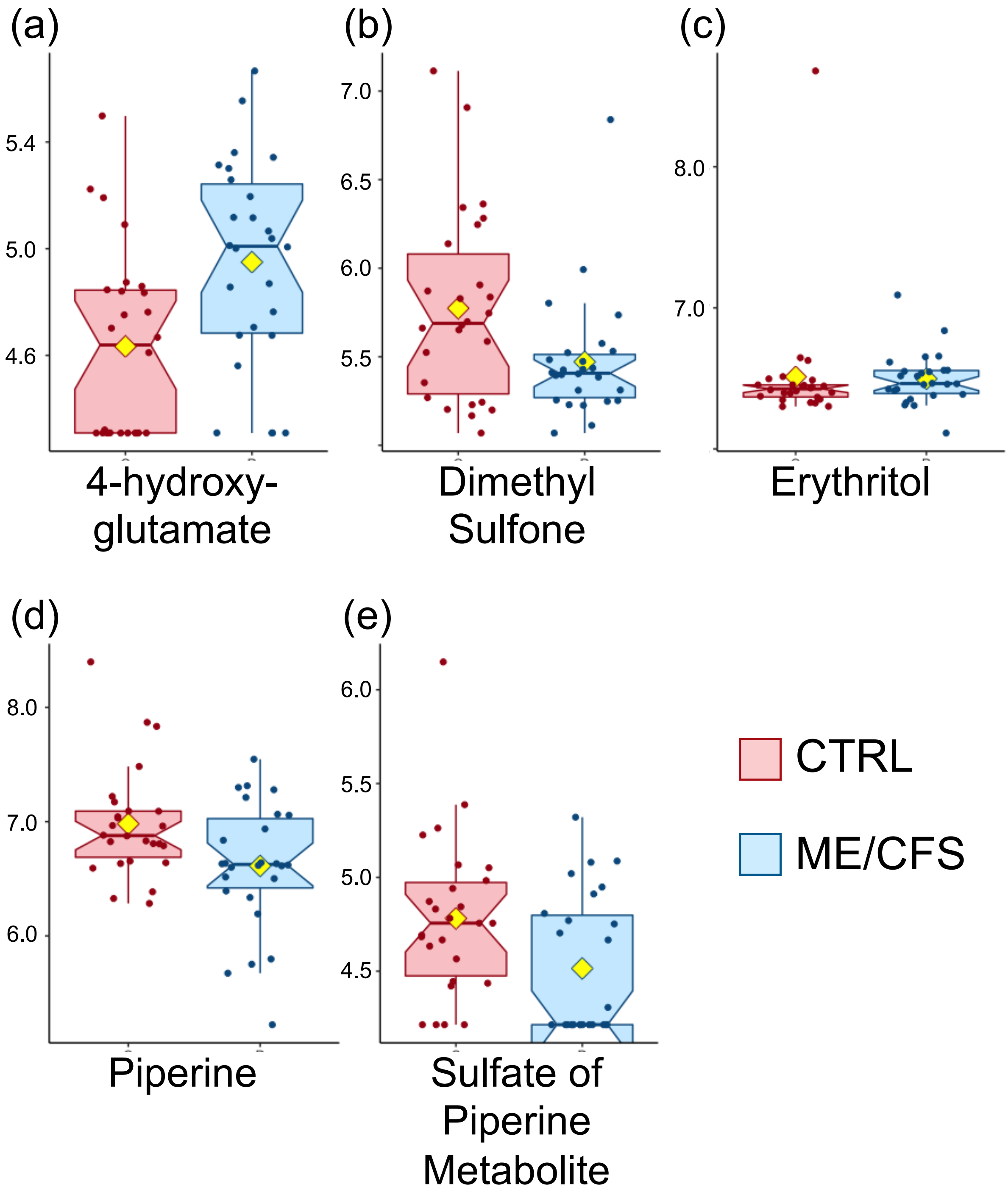

Figure S1: Box plot distribution of logged values for the metabolites in Table 2 that are not part of the Acyl Choline pathway. Controls (CTRL) are shown in red and patients (ME/CFS) in blue. The yellow diamond represents the mean.

Figure S2: ChemRICH sub-pathway-based enrichment plot.

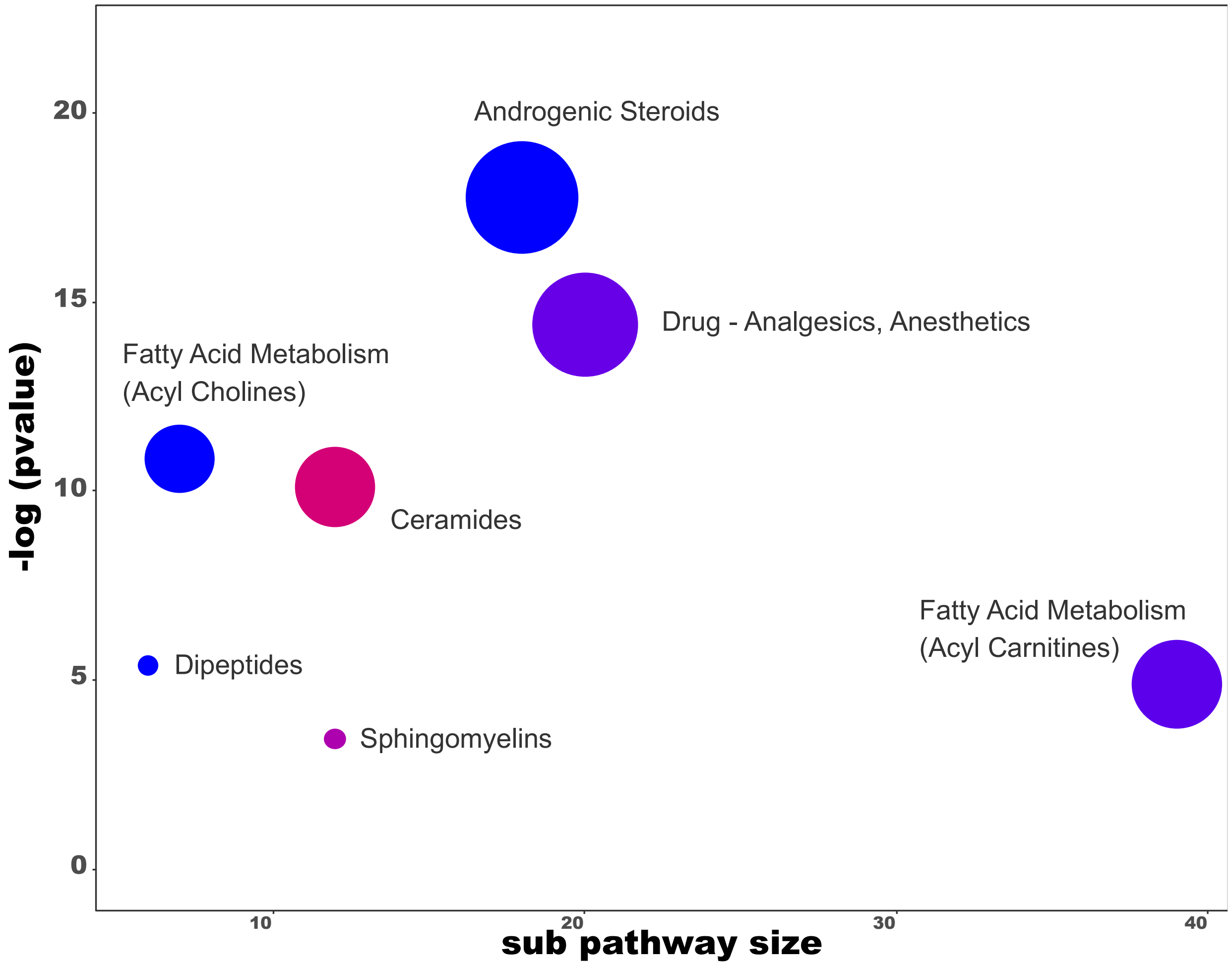

Clusters were generated based on Metabolon®'s classification.

Cluster colors give the proportion of increased or decreased compounds (red = increased, blue = decreased). Enrichment statistics are calculated by Kolmogorov-Smirnov test.

Enrichment clusters with significant differences at $p < 0.05$ are shown.

Figure S3: Display of the fold change (controls/patients) of the median, averaged for each of 89 sub-pathways from Germain at al. (2018).

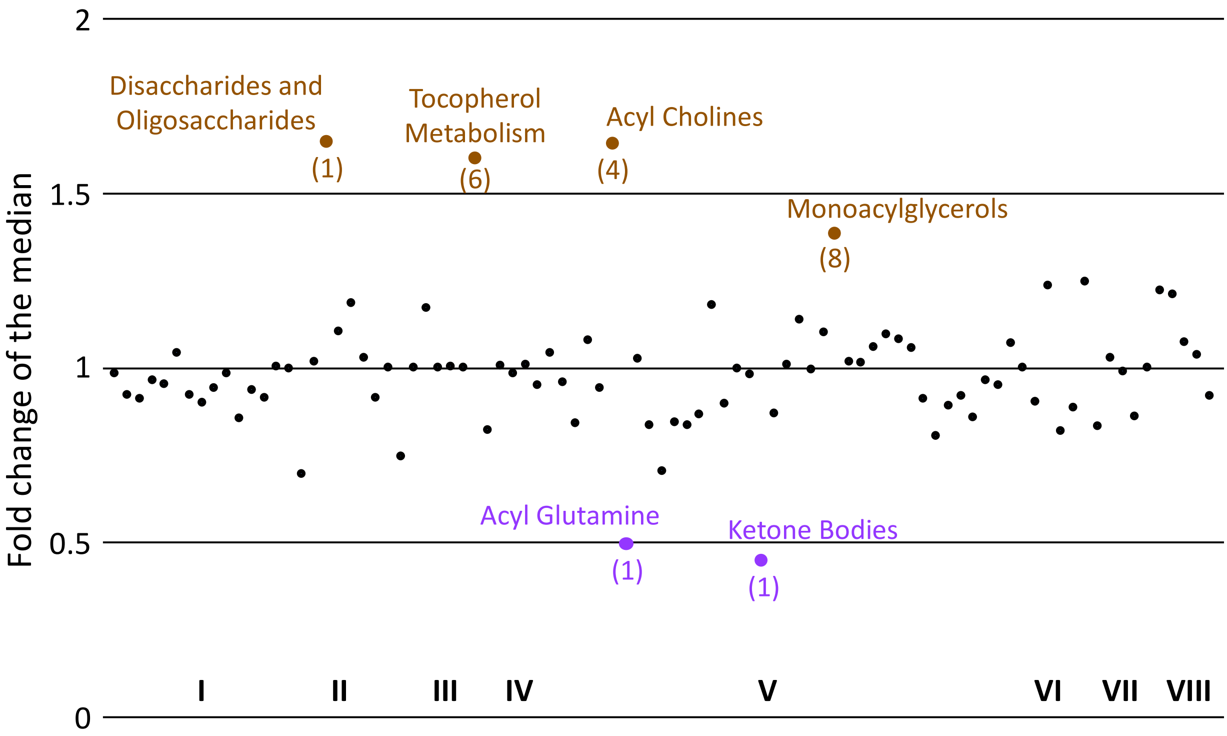

Roman numbers at the bottom of the figure are assigned as follow for each of the nine super-pathways: I = Amino Acids, II = Carbohydrates, III = Cofactors and Vitamins, IV = Energy, V = Lipids, VI = Nucleotides, VII = Peptides and VIII = Xenobiotics. Labelled sub-pathways are discussed in the manuscript; brown ones are over-abundant in controls compared to patients while purple ones are the opposite. The number associated with each sub-pathway reflects the number of metabolites included. Omitted from the graph are the Drug sub-pathways as well as the Tobacco Metabolites, all classified as Xenobiotics.

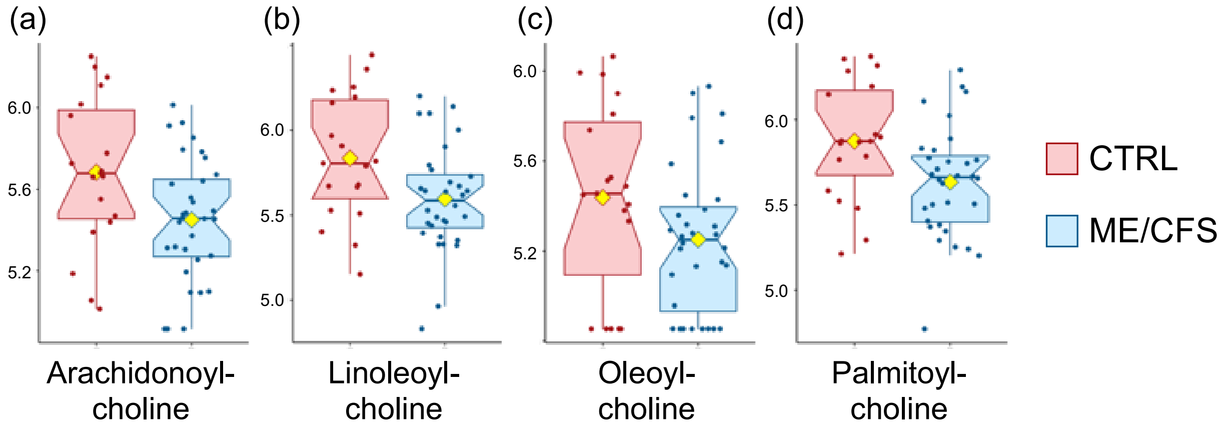

Figure S4: Box plot distribution of logged values for the metabolites that are part of the Acyl Choline pathway from Germain et al. (2018). Controls (CTRL) are shown in red and patients (ME/CFS) in blue. The yellow diamond represents the mean.

Table S1: List of metabolites found to be significantly different between controls and patients after Wilcoxon rank-sum testing with a *p*-value cutoff of 0.05.

Super-pathway	Sub-pathway	Metabolite	HMDB ID	Fold change	*p*-value
Amino Acids	Alanine and Aspartate Metabolism	N-carbamoylalanine	NA	0.6	0.049
	Glutamate Metabolism	4-hydroxyglutamate	HMDB01344	0.5	0.004
	Glutathione Metabolism	Cysteinylglycine	HMDB00078	1	0.008
	Histidine Metabolism	N-acetyl-1-methylhistidine*	NA	0.7	0.038
	Methionine, Cysteine, SAM and Taurine Metabolism	Hypotaurine	HMDB00965	1.3	0.039
	Tryptophan Metabolism	Indolelactate	HMDB00671	1.3	0.006
		Tryptophan betaine	HMDB61115	1.7	0.035
	Urea cycle; Arginine and Proline Metabolism	N,N,N-trimethyl-alanylproline betaine (TMAP)	NA	0.9	0.039
Lipids	Androgenic Steroids	Dehydroepiandrosterone sulfate (DHEA-S)	HMDB01032	1.5	0.035
		Epiandrosterone sulfate	NA	1.8	0.035
		Androstenediol (3alpha, 17alpha) monosulfate (2)	NA	1.3	0.039
		5alpha-androstan-3beta,17alpha-diol disulfate	NA	1.7	0.04
		Androsterone sulfate	HMDB02759	1.6	0.041
		Androstenediol (3beta,17beta) disulfate (2)	HMDB03818	1.3	0.044
		Etiocholanolone glucuronide	HMDB04484	1.8	0.049
	Corticosteroids	Cortisone	HMDB02802	1.3	0.004
		Cortisol	HMDB00063	1.3	0.02
	Fatty Acid Metabolism (Acyl Choline)	Dihomo-linolenoyl-choline	NA	2.3	0.019
		Linoleoylcholine*	NA	2.2	0.036
		Stearoylcholine*	NA	2.2	0.043
	Fatty Acid Metabolism(Acyl Carnitine)	Arachidoylcarnitine (C20)*	HMDB06460	0.7	0.025
		Docosahexaenoylcarnitine (C22:6)*	NA	1.3	0.025
		Adrenoylcarnitine (C22:4)*	NA	1.3	0.029
		Octanoylcarnitine (C8)	HMDB00791	1.3	0.035
		Adipoylcarnitine (C6-DC)	HMDB61677	0.8	0.042
		Decanoylcarnitine (C10)	HMDB00651	1.4	0.049
	Fatty Acid, Dicarboxylate	Branched chain 14:0 dicarboxylic acid**	NA	1.5	0.049
	Fatty Acid, Monohydroxy	3-hydroxymyristate	NA	1.4	0.013
		3-hydroxydecanoate	HMDB02203	1.5	0.015
		3-hydroxylaurate	HMDB00387	1.5	0.029
	Medium Chain Fatty Acid	Cis-4-decenoate (10:1n6)*	NA	1.5	0.047
	Secondary Bile Acid Metabolism	Glycohyocholate	NA	0.7	0.048
	Short Chain Fatty Acid	Valerate (5:0)	HMDB00892	1.4	0.045
Peptides	Dipeptide	Phenylalanylalanine	HMDB028988	1.3	0.007
		Phenylalanylglycine	HMDB28995	1.3	0.014
		Valylleucine	HMDB29131	1.5	0.046
	Gamma-glutamyl Amino Acid	Gamma-glutamyltyrosine	HMDB11741	0.8	0.045
Xenobiotics	Chemical	Dimethyl sulfone	HMDB04983	2.7	0.032
	Food Component/Plant	Piperine	HMDB29377	3	0.027
		Sulfate of piperine metabolite C16H19NO3 (3)*	NA	2.5	0.027
		Stachydrine	HMDB04827	1.6	0.044

HMDB stands for Human Metabolome Database. NA stands for Not Assigned. Fold change represents controls/patients and red highlights the eight compounds that have a higher abundance in patients compared to controls.

Table S2: List of metabolites with a fold change (controls/patients) threshold of 2.

Super-pathway	Sub-pathway	Metabolite	HMDB ID	Fold change
Amino Acids	Glutamate Metabolism	4-hydroxyglutamate	HMDB01344	0.5
	Glycine, Serine and Threonine Metabolism	O-acetylhomoserine	NA	3.1
	Tryptophan Metabolism	Picolinate	HMDB02243	2.7
Carbohydrates	Fructose, Mannose and Galactose Metabolism	Galactonate	HMDB00565	0.5
Cofactors and Vitamins	Vitamin B6 Metabolism	Pyridoxate	HMDB00017	3.1
Lipids	Fatty Acid Metabolism (Acyl Choline)	Oleoylcholine	NA	2.3
		Dihomo-linolenoyl-choline	NA	2.3
		Linoleoylcholine	NA	2.2
		Stearoylcholine	NA	2.2
		Palmitoylcholine	NA	2.1
	Primary Bile Acid Metabolism	Cholate	HMDB00619	3.1
Peptides	Fibrinogen Cleavage Peptide	Fibrinopeptide B (1-11)**	NA	3.9
		Fibrinopeptide A (2-15)**	NA	2.5
Xenobiotics	Benzoate Metabolism	3-(3-hydroxyphenyl)propionate	HMDB00375	0.4
		2-hydroxyhippurate (salicylurate)	HMDB00840	2.3
		3-hydroxyhippurate	HMDB06116	0.5
	Chemical	4-methylbenzenesulfonate	NA	58
		Dimethyl sulfone	HMDB04983	2.7
	Drug - Analgesics, Anesthetics	Ibuprofen	HMDB01925	0.1
	Food Component/Plant	Erythritol	HMDB02994	6
		Thymol sulfate	HMDB01878	4.4
		Piperine	HMDB29377	3
		Eugenol sulfate	NA	2.8
		S-allylcysteine	HMDB34323	2.6
		Sulfate of piperine metabolite C16H19NO3 (3)*	NA	2.5

HMDB stands for Human Metabolome Database. NA stands for Not Assigned. Fold changes in red lettering highlight the five compounds that have a higher abundance in patients compared to controls.

Table S3: List of metabolites found to be significantly different between controls and patients after Wilcoxon rank-sum testing on super-pathway dichotomized datasets, with a *q*-value cutoff at 0.15.

**Super-pathway**	**Sub-pathway**	**Metabolite**	**HMDB ID**	***p*-value**	***q*-value**	**Fold change**
Peptides	Dipeptides	Leucylglycine	HMDB28929	0.003	0.1	2
		Phenylalanylalanine	HMDB028988	0.007	0.11	1.3
		Phenylalanylglycine	HMDB28995	0.01	0.15	1.3

HMDB stands for Human Metabolome Database.

Fold change of mean represents controls/patients.

Table S4: Results of the pathway enrichment analysis on the global metabolomics dataset.

Pathway name	Match status	*p*-value	*q*-value	Impact score
Glutathione metabolism	3/38	0.01	0.34	0.01
Steroid hormone biosynthesis	8/99	0.01	0.34	0.08
Propanoate metabolism	3/35	0.04	0.50	0.03
Terpenoid backbone biosynthesis	1/33	0.07	0.50	0
Pentose phosphate pathway	3/32	0.07	0.50	0.11
Ascorbate and aldarate metabolism	5/45	0.07	0.50	0.06
Glycine, serine and threonine metabolism	9/48	0.08	0.50	0.06
Glycolysis or Gluconeogenesis	2/31	0.08	0.50	0.10
Inositol phosphate metabolism	2/39	0.08	0.50	0.14
Vitamin B6 metabolism	2/32	0.08	0.50	0.08
D-Glutamine and D-glutamate metabolism	1/11	0.10	0.57	0.11
Alanine, aspartate and glutamate metabolism	3/24	0.11	0.58	0.44
Valine, leucine and isoleucine biosynthesis	7/27	0.15	0.61	0.26
Pentose and glucuronate interconversions	6/53	0.16	0.61	0.12
Pyruvate metabolism	4/32	0.18	0.61	0.32
Citrate cycle (TCA cycle)	2/20	0.19	0.61	0.13
Arachidonic acid metabolism	1/62	0.19	0.61	0.22
Butanoate metabolism	4/40	0.21	0.61	0.11
Taurine and hypotaurine metabolism	6/20	0.21	0.61	0.51
Cysteine and methionine metabolism	11/56	0.21	0.61	0.36
Lysine biosynthesis	2/32	0.22	0.61	0.07
Amino sugar and nucleotide sugar metabolism	5/88	0.23	0.61	0.01
Fructose and mannose metabolism	1/48	0.24	0.61	0.01
Glyoxylate and dicarboxylate metabolism	5/50	0.25	0.61	0.20
Sphingolipid metabolism	4/25	0.25	0.61	0.26
Tryptophan metabolism	7/79	0.26	0.61	0.15
Porphyrin and chlorophyll metabolism	4/104	0.29	0.65	0.06
Arginine and proline metabolism	14/77	0.34	0.73	0.36
Starch and sucrose metabolism	6/50	0.35	0.73	0.11
Histidine metabolism	10/44	0.36	0.73	0.15
Ether lipid metabolism	2/23	0.40	0.75	0
Nicotinate and nicotinamide metabolism	10/44	0.42	0.75	0.09
Pantothenate and CoA biosynthesis	7/27	0.44	0.75	0.24
Lysine degradation	7/47	0.45	0.75	0.12
Phenylalanine metabolism	12/45	0.45	0.75	0.19
alpha-Linolenic acid metabolism	1/29	0.45	0.75	0
Primary bile acid biosynthesis	8/47	0.45	0.75	0.10
Glycerophospholipid metabolism	7/39	0.47	0.75	0.31
Nitrogen metabolism	6/39	0.55	0.83	0
Purine metabolism	5/92	0.55	0.83	0.14
Cyanoamino acid metabolism	1/16	0.58	0.83	0
Fatty acid elongation in mitochondria	1/27	0.58	0.83	0
beta-Alanine metabolism	5/28	0.59	0.83	0.07
Galactose metabolism	6/41	0.65	0.88	0.10
Methane metabolism	1/34	0.68	0.88	0
Aminoacyl-tRNA biosynthesis	6/75	0.68	0.88	0.11
D-Arginine and D-ornithine metabolism	2/8	0.68	0.88	0
Tyrosine metabolism	9/76	0.70	0.89	0.22
Pyrimidine metabolism	10/60	0.73	0.91	0.21
Retinol metabolism	2/22	0.75	0.91	0.35
Ubiquinone and other terpenoid-quinone biosynthesis	3/36	0.77	0.91	0.09
Caffeine metabolism	10/21	0.79	0.91	0.64
Valine, leucine and isoleucine degradation	3/40	0.80	0.91	0.06
Linoleic acid metabolism	4/15	0.82	0.91	0.66
Phenylalanine, tyrosine and tryptophan biosynthesis	3/27	0.83	0.91	0.01
Fatty acid metabolism	2/50	0.85	0.91	0.03
Glycerolipid metabolism	4/32	0.87	0.91	0.23
Fatty acid biosynthesis	6/49	0.87	0.91	0
Riboflavin metabolism	3/21	0.88	0.91	0.25
Synthesis and degradation of ketone bodies	1/6	0.94	0.95	0
Thiamine metabolism	1/24	0.97	0.97	0

Match status represents the number of hits compared to the number of metabolites in the selected pathway, while the impact score indicates the pathway impact on the health of the subjects based the affected metabolites within the pathway as well as the abundance differences between controls and patients.

Table S5: Results of the pathway enrichment analysis on the complex lipid dataset.

Pathway name	Match status	*p*-value	*q*-value	Impact score
Sphingolipid metabolism	4/25	0.03	0.23	0.34
Fatty acid metabolism	1/50	0.19	0.52	0.03
Fatty acid elongation in mitochondria	1/27	0.19	0.52	0.00
Fatty acid biosynthesis	6/49	0.65	0.87	0.00
alpha-Linolenic acid metabolism	2/29	0.65	0.87	0.00
Arachidonic acid metabolism	2/62	0.79	0.87	0.22
Linoleic acid metabolism	2/15	0.86	0.87	0.66
Glycerophospholipid metabolism	2/39	0.87	0.87	0.10

Match status represents the number of hits compared to the number of metabolites in the selected pathway, while the impact score indicates the pathway impact on the health of the subjects based the affected metabolites within the pathway as well as the abundance differences between controls and patients.

Table S6: ChemRICH output for the Global Metabolomics panel analysis.

Cluster name	Cluster size	*p*-value	*q*-value	Key compound	Altered metabolites	Increased	Decreased	Increased ratio	Altered ratio
Dipeptides	52	0.025	1	4-hydroxyglutamate	9	6	3	0.7	0.2
NewCluster_12	6	0.057	1	arachidoylcarnitine (C20)*	3	1	2	0.3	0.5
Indoles	7	0.15	1	indolelactate	2	2	0	1	0.3
Pipecolic Acids	5	0.21	1	C-glycosyltryptophan	2	1	1	0.5	0.4
Pyrrolidinones	11	0.22	1	cotinine	2	2	0	1	0.2
UnSaturated FA	26	0.34	1	3-(cystein-S-yl)acetaminophen*	2	1	0	0.5	0.08
Xanthines	15	0.74	1	leucylglycine	2	2	0	1	0.1
4-Hydroxycoumarins	3	1	1	nonanoylcarnitine (C9)	0	0	0	ND	0
Acetanilides	8	1	1	4-acetaminophen sulfate	0	0	0	ND	0
Adenosine	4	1	1	5-methylthioadenosine (MTA)	0	0	0	ND	0
Adipates	5	1	1	threonate	0	0	0	ND	0
Amino Acids	12	1	1	5,6-dihydrothymine	0	0	0	ND	0
Amino Acids, Aromatic	6	1	1	phenylalanine	0	0	0	ND	0
Amino Acids, Basic	11	1	1	dimethyl sulfone	1	1	0	1	0.09
Amino Acids, Branched-Chain	4	1	1	4-methylbenzenesulfonate	0	0	0	ND	0
Amino Acids, Diamino	4	1	1	2-hydroxyphytanate*	0	0	0	ND	0
Amino Acids, Sulfur	5	1	1	N-acetylarginine	0	0	0	ND	0
Androstenols	4	1	1	3-methylglutarate/2-methylglutarate	0	0	0	ND	0
Anisoles	4	1	1	phenylacetylcarnitine	0	0	0	ND	0
Arachidonic Acids	3	1	1	arachidonate (20:4n6)	0	0	0	ND	0
Bile Pigments	4	1	1	3-amino-2-piperidone	0	0	0	ND	0
Butyrates	9	1	1	alpha-ketobutyrate	0	0	0	ND	0
Caproates	7	1	1	3beta-hydroxy-5-cholestenoate	0	0	0	ND	0
Carnitine	19	1	1	oleoylcholine	1	1	0	1	0.05
Chenodeoxycholic Acid	3	1	1	glycochenodeoxycholate	0	0	0	ND	0
Cholestenones	5	1	1	4-cholesten-3-one	0	0	0	ND	0
Cholic Acids	8	1	1	phenylacetylglutamine	0	0	0	ND	0
Cinnamates	5	1	1	4-ethylphenylsulfate	0	0	0	ND	0
Cresols	7	1	1	p-cresol glucuronide*	0	0	0	ND	0
Deoxycholic Acid	5	1	1	alpha-hydroxymetoprolol	1	0	1	0	0.2
Dicarboxylic Acids	7	1	1	tartronate (hydroxymalonate)	0	0	0	ND	0
Ethanolamines	4	1	1	phosphoethanolamine	0	0	0	ND	0
Fluorocarbons	3	1	1	perfluorooctanoate (PFOA)	0	0	0	ND	0
Gluconates	4	1	1	gluconate	0	0	0	ND	0
Glucuronates	4	1	1	palmitoylcholine	0	0	0	ND	0
Glutamates	7	1	1	glutamate	0	0	0	ND	0
Glutarates	13	1	1	4-hydroxy-2-oxoglutaric acid	0	0	0	ND	0
Glycerophospholipids	3	1	1	glycerophosphoethanolamine	0	0	0	ND	0
Guanidines	5	1	1	androsterone sulfate	1	1	0	1	0.2
Hexoses	3	1	1	glucose	0	0	0	ND	0
Hippurates	4	1	1	hippurate	0	0	0	ND	0
Hydroquinones	4	1	1	5-dodecenoylcarnitine (C12:1)	0	0	0	ND	0
Hydroxybenzoates	3	1	1	salicylate	0	0	0	ND	0
Hydroxyproline	4	1	1	androsterone glucuronide	0	0	0	ND	0
Imidazoles	9	1	1	1-methyl-4-imidazoleacetate	0	0	0	ND	0
Imino Acids	3	1	1	21-hydroxypregnenolone disulfate	0	0	0	ND	0
Keto Acids	3	1	1	4-methyl-2-oxopentanoate	0	0	0	ND	0
Lauric Acids	3	1	1	decanoylcarnitine (C10)	1	1	0	1	0.3
Lysophospholipids	4	1	1	2-methoxyacetaminophen glucuronide*	1	1	0	1	0.2
Malates	3	1	1	2-isopropylmalate	0	0	0	ND	0
Malonates	3	1	1	methylmalonate (MMA)	0	0	0	ND	0
Methionine	5	1	1	bromine	0	0	0	ND	0
Methylhistidines	4	1	1	N-acetyl-1-methylhistidine*	1	0	1	0	0.2
Naphthalenes	3	1	1	nisinate (24:6n3)	0	0	0	ND	0
O=FA_20_1	3	1	1	suberate (C8-DC)	0	0	0	ND	0
O=FA_9_1	5	1	1	dehydroepiandrosterone sulfate (DHEA-S)	1	1	0	1	0.2
OH-FA_14_0_1	6	1	1	N-ethylglycinexylidide	0	0	0	ND	0
OH-FA_22_0_1	5	1	1	7-methylurate	0	0	0	ND	0
OH-FA_8_0_1	3	1	1	4-acetamidobutanoate	0	0	0	ND	0
Oleic Acids	3	1	1	oleoyl ethanolamide	0	0	0	ND	0
Pentanoic Acids	7	1	1	valerate (5:0)	1	1	0	1	0.1
Pentoses	4	1	1	N-acetylserine	0	0	0	ND	0
Phenoxypropanolamines	3	1	1	metoprolol	0	0	0	ND	0
Phenylacetates	5	1	1	4-acetamidophenylglucuronide	0	0	0	ND	0
Phenylpropionates	3	1	1	N2,N5-diacetylornithine	0	0	0	ND	0
Phytosterols	4	1	1	cortisone	1	1	0	1	0.2
Pregnenolone	3	1	1	pregnenolone sulfate	0	0	0	ND	0
Propanolamines	3	1	1	glycohyocholate	1	0	1	0	0.3
Pyridines	7	1	1	nicotinamide	0	0	0	ND	0
Pyridones	3	1	1	2-hydroxystearate	0	0	0	ND	0
Pyrimidinones	6	1	1	cytosine	0	0	0	ND	0
Pyrroles	3	1	1	stachydrine	1	1	0	1	0.3
Pyruvates	5	1	1	phenyllactate (PLA)	0	0	0	ND	0
Quinolines	4	1	1	imidazole lactate	0	0	0	ND	0
Retinoids	3	1	1	glucuronate	0	0	0	ND	0
Saturated FA	17	1	1	3-hydroxydecanoate	1	1	0	1	0.06
Saturated_Fatty Acids	6	1	1	3-hydroxymyristate	1	1	0	1	0.2
Succinates	6	1	1	androstenediol (3beta,17beta) disulfate (2)	1	1	0	1	0.2
Sugar Acids	8	1	1	picolinoylglycine	0	0	0	ND	0
Sugar Alcohols	5	1	1	erythritol	0	0	0	ND	0
Sulfur Compounds	4	1	1	N-carbamoylalanine	1	0	1	0	0.2
Sulfuric Acid Esters	5	1	1	leucylglutamine*	0	0	0	ND	0
Taurodeoxycholic Acid	3	1	1	taurochenodeoxycholate	0	0	0	ND	0
Thiazoles	3	1	1	saccharin	0	0	0	ND	0
Tricarboxylic Acids	6	1	1	gamma-carboxyglutamate	0	0	0	ND	0
Trimethyl Ammonium Compounds	4	1	1	dimethylglycine	0	0	0	ND	0
Uridine	7	1	1	10-undecenoate (11:1n1)	0	0	0	ND	0

ND stands for Not Determined. Fold change represents controls/patients.

Table S7: ChemRICH output for the Dipeptides cluster name.

**Super-pathway**	**Sub-pathway**	**Metabolite**	**HMDB ID**	**Fold change**	***p*-value**
Amino Acids	Glutamate Metabolism	4-hydroxyglutamate	HMDB01344	0.5	0.005
	Alanine and Aspartate Metabolism	Hydroxyasparagine	HMDB32332	0.8	0.007
	Glutathione Metabolism	Cysteinylglycine	HMDB00078	1.4	0.008
Peptides	Dipeptides	Phenylalanylalanine	HMDB028988	1.3	0.007
		Phenylalanylglycine	HMDB28995	1.3	0.014
		Valylleucine	HMDB29131	1.5	0.046
	Gamma-glutamyl Amino Acid	Gamma-glutamyltyrosine	HMDB11741	0.8	0.045
Lipids	Fatty Acid, Monohydroxy	3-hydroxylaurate	HMDB00387	1.5	0.029
	Androgenic Steroids	Etiocholanolone glucuronide	HMDB04484	1.8	0.049

HMDB stands for Human Metabolome Database.
Fold change of mean represents controls/patients.

Table S8: ChemRICH output for the complex lipid panel analysis.

Cluster name	Cluster size	*p*-value	*q*-value	Key compound	Altered metabolites	Increased	Decreased	Increased ratio	Altered ratio
Unsaturated_Ceramides	9	0.00036	0.0072	CER(18:0)	3	0	3	0	0.3
Sphingomyelins	9	0.035	0.35	SM(18:1)	2	0	2	0	0.2
Cholesterol Esters	21	1	1	CE(22:2)	1	0	1	0	0.05
Galactosylceramides	8	1	1	HCER(20:0)	1	0	1	0	0.1
Lactosylceramides	9	1	1	LCER(18:0)	0	0	0	ND	0
NewCluster_4	30	1	1	PE(P-18:1/16:0)	0	0	0	ND	0
NewCluster_6	12	1	1	LPE(20:1)	0	0	0	ND	0
Plasmalogens	6	1	1	PE(P-18:1/22:6)	0	0	0	ND	0
Saturated_Ceramides	7	1	1	DCER(20:0)	0	0	0	ND	0
Saturated_Diglycerides	5	1	1	DAG(16:0/18:0)	0	0	0	ND	0
Saturated FA	9	1	1	FFA(24:0)	0	0	0	ND	0
Saturated_Lysophosphatidylcholines	5	1	1	LPC(15:0)	0	0	0	ND	0
Saturated_Lysophospholipids	4	1	1	LPE(15:0)	0	0	0	ND	0
Saturated_Phosphatidylcholines	7	1	1	PC(16:0/14:0)	0	0	0	ND	0
Saturated_Phosphatidylethanolamines	3	1	1	PE(16:0/16:0)	0	0	0	ND	0
Unsaturated_Diglycerides	49	1	1	DAG(18:1/18:1)	0	0	0	ND	0
UnSaturated FA	14	1	1	FFA(22:4)	0	0	0	ND	0
Unsaturated_Lysophosphatidylcholines	10	1	1	LPC(18:3)	0	0	0	ND	0
Unsaturated_Phosphatidylcholines	80	1	1	PC(18:2/18:3)	1	1	0	1	0.01
Unsaturated_Phosphatidylethanolamines	49	1	1	PE(18:1/14:1)	0	0	0	ND	0

ND stands for Not Determined. Fold change represents controls/patients.

Table S9: Detailed ChemRICH output for the sub-pathway-based analysis.

Cluster name	Cluster size	p-value	q-value	Altered metabolites	Increased	Decreased	Increased ratio	Altered ratio
Androgenic Steroids	18	0.000000019	0.0000021	11	0	11	0	0.6
Drug - Analgesics, Anesthetics	20	0.00000055	0.00003	10	2	8	0.1	0.5
Fatty Acid Metabolism (Acyl Choline)	7	0.000019	0.0007	6	0	6	0	0.9
Ceramide	12	0.000041	0.0011	7	7	0	0.6	0.6
Dipeptide	6	0.0046	0.1	4	0	4	0	0.7
Fatty Acid Metabolism(Acyl Carnitine)	39	0.0076	0.14	8	3	5	0.08	0.2
Sphingomyelin	12	0.032	0.5	4	4	0	0.3	0.3
Corticosteroids	4	0.056	0.77	2	0	2	0	0.5
Gamma-glutamyl Amino Acid	16	0.15	1	5	5	0	0.3	0.3
Fatty Acid, Monohydroxy	19	0.15	1	4	0	4	0	0.2
Food Component/Plant	49	0.28	1	10	2	8	0.04	0.2
Tryptophan Metabolism	19	0.36	1	4	1	3	0.05	0.2
Alanine and Aspartate Metabolism	10	0.55	1	2	2	0	0.2	0.2
Acetylated Peptides	4	1	1	1	1	0	0.2	0.2
Aminosugar Metabolism	5	1	1	1	1	0	0.2	0.2
Ascorbate and Aldarate Metabolism	3	1	1	1	1	0	0.3	0.3
Bacterial/Fungal	3	1	1	0	0	0	0	0
Benzoate Metabolism	21	1	1	1	1	0	0.05	0.05
CE Ester	26	1	1	7	7	0	0.3	0.3
Chemical	23	1	1	1	0	1	0	0.04
Creatine Metabolism	3	1	1	0	0	0	0	0
DAG Ester	58	1	1	0	0	0	0	0
Dihydroceramide	13	1	1	1	1	0	0.08	0.08
Drug - Cardiovascular	12	1	1	1	1	0	0.08	0.08
Drug - Gastrointestinal	4	1	1	0	0	0	0	0
Drug - Neurological	5	1	1	0	0	0	0	0
Drug - Psychoactive	12	1	1	1	1	0	0.08	0.08
Drug - Respiratory	4	1	1	2	2	0	0.5	0.5
Drug - Topical Agents	3	1	1	0	0	0	0	0
Endocannabinoid	5	1	1	0	0	0	0	0
Ester	26	1	1	2	0	2	0	0.08
Fatty Acid Metabolism (also BCAA Metabolism)	4	1	1	1	1	0	0.2	0.2
Fatty Acid Metabolism(Acyl Glycine)	3	1	1	0	0	0	0	0
Fatty Acid, Amino	3	1	1	0	0	0	0	0
Fatty Acid, Branched	5	1	1	0	0	0	0	0
Fatty Acid, Dicarboxylate	28	1	1	3	2	1	0.07	0.1
Fatty Acid, Dihydroxy	6	1	1	0	0	0	0	0
Fibrinogen Cleavage Peptide	7	1	1	0	0	0	0	0
Fructose, Mannose and Galactose Metabolism	4	1	1	0	0	0	0	0
Glutamate Metabolism	14	1	1	1	1	0	0.07	0.07
Glutathione Metabolism	7	1	1	2	0	2	0	0.3
Glycerolipid Metabolism	3	1	1	0	0	0	0	0
Glycine, Serine and Threonine Metabolism	11	1	1	2	0	2	0	0.2
Glycolysis, Gluconeogenesis, and Pyruvate Metabolism	6	1	1	0	0	0	0	0
Hemoglobin and Porphyrin Metabolism	5	1	1	0	0	0	0	0
Hexosylceramide	12	1	1	3	3	0	0.2	0.2
Histidine Metabolism	15	1	1	1	1	0	0.07	0.07
Lactosylceramide	12	1	1	2	2	0	0.2	0.2
Leucine, Isoleucine and Valine Metabolism	31	1	1	1	1	0	0.03	0.03
Long Chain Fatty Acid	13	1	1	1	0	1	0	0.08
LPC Ester	18	1	1	0	0	0	0	0
LPE Ester	17	1	1	1	1	0	0.06	0.06
Lysine Metabolism	15	1	1	1	1	0	0.07	0.07
Lysophospholipid	6	1	1	0	0	0	0	0
Medium Chain Fatty Acid	9	1	1	1	0	1	0	0.1
Methionine, Cysteine, SAM and Taurine Metabolism	22	1	1	2	1	1	0.05	0.09
Monounsaturated FFA	6	1	1	0	0	0	0	0
Nicotinate and Nicotinamide Metabolism	7	1	1	1	0	1	0	0.1
PC Ester	103	1	1	2	1	1	0.01	0.02
PE Ester	61	1	1	0	0	0	0	0
PE Ether	20	1	1	0	0	0	0	0
PE Plasmalogen	46	1	1	0	0	0	0	0
Pentose Metabolism	7	1	1	0	0	0	0	0
Phenylalanine Metabolism	8	1	1	1	0	1	0	0.1
Phospholipid Metabolism	6	1	1	0	0	0	0	0
PI Ester	28	1	1	0	0	0	0	0
Polyamine Metabolism	6	1	1	0	0	0	0	0
Polyunsaturated Fatty Acid (n3 and n6)	16	1	1	1	0	1	0	0.06
Polyunsaturated FFA (PUFA)	10	1	1	0	0	0	0	0
Pregnenolone Steroids	6	1	1	0	0	0	0	0
Primary Bile Acid Metabolism	9	1	1	2	0	2	0	0.2
Progestin Steroids	6	1	1	0	0	0	0	0
Purine Metabolism, (Hypo)Xanthine/Inosine containing	6	1	1	0	0	0	0	0
Purine Metabolism, Adenine containing	4	1	1	0	0	0	0	0
Pyrimidine Metabolism, Cytidine containing	5	1	1	0	0	0	0	0
Pyrimidine Metabolism, Orotate containing	3	1	1	0	0	0	0	0
Pyrimidine Metabolism, Uracil containing	11	1	1	0	0	0	0	0
Saturated FFA	9	1	1	1	0	1	0	0.1
Secondary Bile Acid Metabolism	20	1	1	4	2	2	0.1	0.2
Sphingosines	3	1	1	0	0	0	0	0
Sterol	7	1	1	1	0	1	0	0.1
TAG Ester_FA12:0	17	1	1	0	0	0	0	0
TAG Ester_FA14:0	32	1	1	0	0	0	0	0
TAG Ester_FA14:1	12	1	1	0	0	0	0	0
TAG Ester_FA15:0	14	1	1	0	0	0	0	0
TAG Ester_FA16:0	65	1	1	0	0	0	0	0
TAG Ester_FA16:1	37	1	1	0	0	0	0	0
TAG Ester_FA17:0	13	1	1	0	0	0	0	0
TAG Ester_FA18:0	37	1	1	0	0	0	0	0
TAG Ester_FA18:1	67	1	1	0	0	0	0	0
TAG Ester_FA18:2	55	1	1	0	0	0	0	0
TAG Ester_FA18:3	29	1	1	1	1	0	0.03	0.03
TAG Ester_FA20:0	9	1	1	0	0	0	0	0
TAG Ester_FA20:1	12	1	1	0	0	0	0	0
TAG Ester_FA20:2	12	1	1	0	0	0	0	0
TAG Ester_FA20:3	16	1	1	0	0	0	0	0
TAG Ester_FA20:4	31	1	1	0	0	0	0	0
TAG Ester_FA20:5	13	1	1	0	0	0	0	0
TAG Ester_FA22:1	4	1	1	0	0	0	0	0
TAG Ester_FA22:4	9	1	1	0	0	0	0	0
TAG Ester_FA22:5	15	1	1	0	0	0	0	0
TAG Ester_FA22:6	19	1	1	0	0	0	0	0
TCA Cycle	9	1	1	0	0	0	0	0
Tobacco Metabolite	6	1	1	1	0	1	0	0.2
Tocopherol Metabolism	4	1	1	0	0	0	0	0
Tyrosine Metabolism	13	1	1	1	1	0	0.08	0.08
Urea cycle; Arginine and Proline Metabolism	20	1	1	1	1	0	0.05	0.05
Vitamin A Metabolism	6	1	1	0	0	0	0	0
Xanthine Metabolism	15	1	1	0	0	0	0	0

Table S10: Averaged fold change of the median for each of the 94 sub-pathways.

Super-pathway	Sub-pathway	Averaged fold change of the median
Amino Acid	Alanine and Aspartate Metabolism	0.95
	Creatine Metabolism	0.98
	Glutamate Metabolism	0.95
	Glutathione Metabolism	1.14
	Glycine, Serine and Threonine Metabolism	1.05
	Guanidino and Acetamido Metabolism	0.95
	Histidine Metabolism	1.04
	Leucine, Isoleucine and Valine Metabolism	0.97
	Lysine Metabolism	0.98
	Methionine, Cysteine, SAM and Taurine Metabolis	1.02
	Phenylalanine Metabolism	0.99
	Polyamine Metabolism	0.98
	Tryptophan Metabolism	1.10
	Tyrosine Metabolism	0.98
	Urea cycle; Arginine and Proline Metabolism	0.96
Carbohydrate	Advanced Glycation End-product	0.94
	Aminosugar Metabolism	1.01
	Disaccharides and Oligosaccharides	1.19
	Fructose, Mannose and Galactose Metabolism	1.03
	Glycogen Metabolism	0.89
	Glycolysis, Gluconeogenesis, and Pyruvate Metabo	1.04
	Pentose Metabolism	1.01
Cofactors and Vitamins	Ascorbate and Aldarate Metabolism	0.89
	Hemoglobin and Porphyrin Metabolism	0.93
	Nicotinate and Nicotinamide Metabolism	1.09
	Pantothenate and CoA Metabolism	1.13
	Riboflavin Metabolism	1.28
	Tocopherol Metabolism	1.01
	Vitamin A Metabolism	1.01
	Vitamin B6 Metabolism	1.41
Energy	Oxidative Phosphorylation	1.09
	TCA Cycle	0.97
Lipid	Androgenic Steroids	1.66
	Carnitine Metabolism	1.06
	Ceramide PEs	0.92
	Corticosteroids	1.36
	Endocannabinoid	0.93
	Fatty Acid Metabolism (Acyl Choline)	1.93
	Fatty Acid Metabolism (Acyl Glutamine)	0.77
	Fatty Acid Metabolism (also BCAA Metabolism)	0.87
	Fatty Acid Metabolism(Acyl Carnitine)	1.10
	Fatty Acid Metabolism(Acyl Glycine)	0.87
	Fatty Acid Synthesis	0.85
	Fatty Acid, Amino	0.99
	Fatty Acid, Branched	1.30
	Fatty Acid, Dicarboxylate	1.10
	Fatty Acid, Dihydroxy	1.06
	Fatty Acid, Monohydroxy	1.11
	Glycerolipid Metabolism	0.93
	Inositol Metabolism	0.94
	Ketone Bodies	1.08
	Long Chain Fatty Acid	1.07
	Lysophospholipid	0.92
	Medium Chain Fatty Acid	1.20
	Mevalonate Metabolism	0.96
	Phosphatidylglycerol (PG)	1.02
	Phosphatidylserine (PS)	0.81
	Phospholipid Metabolism	1.05
	Polyunsaturated Fatty Acid (n3 and n6)	1.18
	Pregnenolone Steroids	1.29
	Primary Bile Acid Metabolism	1.39
	Progestin Steroids	1.42
	Secondary Bile Acid Metabolism	1.24
	Short Chain Fatty Acid	1.36
	Sphingolipid Synthesis	1.06
	Sphingosines	1.04
	Sterol	1.05
Nucleotide	Purine Metabolism, (Hypo)Xanthine/Inosine conta	1.13
	Purine Metabolism, Adenine containing	1.00
	Purine Metabolism, Guanine containing	1.07
	Pyrimidine Metabolism, Cytidine containing	1.47
	Pyrimidine Metabolism, Orotate containing	0.90
	Pyrimidine Metabolism, Thymine containing	0.92
	Pyrimidine Metabolism, Uracil containing	1.06
Partially Characterized Molecules	Partially Characterized Molecules	1.05
Peptide	Acetylated Peptides	0.89
	Dipeptide	1.49
	Fibrinogen Cleavage Peptide	1.34
	Gamma-glutamyl Amino Acid	0.92
Xenobiotics	Bacterial/Fungal	1.37
	Benzoate Metabolism	1.10
	Chemical	1.06
	Drug - Topical Agents	1.09
	Food Component/Plant	1.21
	Xanthine Metabolism	1.03
	Drug - Analgesics, Anesthetics	ND
	Drug - Antibiotic	ND
	Drug - Cardiovascular	ND
	Drug - Gastrointestinal	ND
	Drug - Metabolic	ND
	Drug - Neurological	ND
	Drug - Psychoactive	ND
	Drug - Respiratory	ND
	Tobacco Metabolite	ND

ND stands for Not Determined. Fold change represents controls/patients. Fold changes in red highlight the sub-pathways with higher abundance in patients compared to controls.

Table S11: Statistical analysis of Monoacylglycerols measured in Germain et al. (2018).

**Super-pathway**	**Sub-pathway**	**Metabolite**	**HMDB ID**	**Fold change**	***p*-value**
Lipids	Monoacyl-glycerols	1-palmitoleoylglycerol (16:1)*	HMDB11565	1.2	0.74
		1-oleoylglycerol (18:1)	HMDB11567	1	0.47
		1-linoleoylglycerol (18:2)	NA	1.3	0.06
		1-linolenoylglycerol (18:3)	HMDB11569	2	0.03
		2-oleoylglycerol (18:1)	HMDB11537	1.3	0.37
		2-linoleoylglycerol (18:2)	HMDB11538	2.1	0.01
		1-dihomo-linolenylglycerol (20:3)	NA	1	0.35
		1-arachidonylglycerol (20:4)	HMDB11549	1.2	0.59

HMDB stands for Human Metabolome Database.
Fold change of median represents controls/patients.
NA stands for Not Assigned.
